# Pathological complete response to neoadjuvant chemotherapy may improve antitumor immune response via reduction of regulatory T cells in muscle-invasive bladder cancer

**DOI:** 10.1038/s41598-024-51273-7

**Published:** 2024-01-16

**Authors:** Daiki Ikarashi, Shigehisa Kitano, Takashi Tsuyukubo, Makiko Yamashita, Tomohiko Matsuura, Shigekatsu Maekawa, Renpei Kato, Yoichiro Kato, Mitsugu Kanehira, Ryo Takata, Tamotsu Sugai, Wataru Obara

**Affiliations:** 1 Department of Urology, Iwate Medical University School of Medicine, Iwate, 028-3695 Japan; 2 Division of Cancer Immunotherapy Development, Department of Advanced Medical Development, The Cancer Institute Hospital of Japanese Foundation for Cancer Research, Tokyo, 135-8550 Japan; 3 Division of Clinical Chemotherapy, Cancer Chemotherapy Center, Japanese Foundation for Cancer Research, Tokyo, 135-8550 Japan; 4 Department of Pathology, Iwate Medical University School of Medicine, Iwate, 028-3695 Japan

**Keywords:** Cancer, Immunology, Oncology, Urology

## Abstract

The prognosis for patients who achieve a pathologic complete response in bladder cancer is excellent, but the association between their prognosis and the tumor microenvironment is unclear. We investigated the tumor immune microenvironment of those with pathological complete response after platinum-based neoadjuvant chemotherapy for cT2-4aN0M0 bladder cancer using multiplex fluorescence immunohistochemistry. Our retrospective study included 12 patients with pathological complete response who underwent radical cystectomy following neoadjuvant chemotherapy for cT2-4aN0M0 muscle-invasive bladder cancer. We assessed the density of several immune cell types in pretreatment and posttreatment tissues via multiplex fluorescence immunohistochemical analysis. The median age was 67 years; 10 patients were male. Nine (75%) and 3 (25%) patients were cT2 and cT3, respectively. The 5-year progression-free and overall survivals were 90% and 100%, respectively. The densities of regulatory T cells (Treg; CD3^+^CD4^+^FoxP3^+^ cell) were significantly decreased and almost disappeared in the tumor microenvironment of posttreatment tissue compared with pretreatment tissue. Other immune cells, such as effector T cells or M2 macrophages, were not significantly changed between posttreatment and pretreatment tissues. In pathological complete response, Tregs in the tumor immune microenvironment were significantly decreased after platinum-based chemotherapy for muscle-invasive bladder cancer. The temporary arresting of immune response in the tumor microenvironment may reflect a favorable prognosis due to the decrease of Tregs with tumor shrinkage and improve the host tumor immune response.

## Introduction

Platinum-based neoadjuvant chemotherapy (NAC) followed by radical cystectomy is the standard treatment for patients with muscle-invasive bladder cancer (MIBC). Pathological complete response (pCR) after NAC is a powerful prognostic indicator of overall survival (OS) for MIBC^1,2^. Patients with pCR to NAC have excellent outcomes, with reported 5-yr OS rates of 80–85%^3^. This appears to be an ideal outcome of NAC. Moreover, previous findings showed that the extent of pT0 following NAC varied between 14 and 38%^3,4^. Recently, the result of a randomized phase III trial of dose-dense methotrexate, vinblastine, doxorubicin, and cisplatin (dd-MVAC) or gemcitabine and cisplatin (GC) as NAC for MIBC has been reported, and pCR was observed in 42% and 36% of patients treated with dd-MVAC and GC, respectively^5^.

The tumor immune microenvironment (TiME consists of tumor cells, immune cells, cytokines, and other components. The interactions between these components, which are antitumor or protumor, determine the trend of antitumor immunity. The immune system can eliminate tumors through the cancer-immune cycle. Conversely, the tumors appear to eventually evade immune surveillance by shaping an immunosuppressive microenvironment^6^. Furthermore, the context of TiME determined at diagnosis reflects the efficacy of chemotherapy^7,8^, and changes in the number of various immune cells infiltrating the TiME are associated with clinical outcomes^7,9^. The transcriptome analysis of pCR cases after cisplatin-based chemotherapy for MIBC robustly expressed fibrosis and extracellular matrix markers, consistent with the wound-healing response that leads to scarring^10,11^. However, the immune gene signature or context of immune cells in pCR cases remains unknown. As previous report, immunotherapy becomes less effective as tumor mass increases, it is thought that immunosuppression intensifies with increasing tumor burden^12^. Immunosuppression by the tumor plays an important role, and surgical resection of primary tumor led to reversal immunosuppression^13^. On the other hand, few reports have focused on chemotherapy-induced tumor shrinkage and changes in immunosuppressive status in the TiME. Therefore, our study aimed to evaluate changes of immunocompetent cells in patients with chemotherapy-induced pCR.

Multiplex fluorescence immunohistochemistry (mFIHC) is a powerful tool for comprehensively analyzing the immune cell type in the tumor microenvironment, compared with traditional immunohistochemistry, as we previously reported^7^. mFIHC enables the quantification of immune cells and a more detailed characterization of each lineage cell^14^. Moreover, we reported that the preexisting TiME predicts the clinical response and prognosis of MIBC in the NAC setting. In the study, intratumoral CD204^+^ cells as M2 macrophages were associated with poor NAC response and prognosis^7^. However, the TiME of pCR cases remains unclear.

Considering this background, we hypothesized that the TiME in pCR cases after chemotherapy might be associated with long-term prognosis. This study assessed the TiME of pCR patients treated with NAC for MIBC using mFIHC. To the best of our knowledge, this is the first study to demonstrate the change in each immune cell in the TiME of pCR cases via mFIHC before and after NAC in MIBC.

## Materials and methods

### Patients

We retrospectively evaluated 12 patients with pCR among 51 who received platinum-based NAC following radical cystectomy at Iwate Medical University Hospital from October 2010 to October 2019. All patients were treated with curative intent. Paired samples were obtained before and after NAC from the same cases. Pre-NAC tissue samples were obtained through transurethral cold cup biopsy or resection, and post-NAC tissue samples were obtained from radical cystectomy in all patients. Tissue samples of pCR were collected from the tumor bed of radical cystectomy specimens. The pCR were assessed by checking for the absence of viable cancer cells in cystectomy specimens which were taken as a whole tissue section. The patients received two or three cycles of platinum-based NAC following radical cystectomy. We also identified NAC non-responder groups with ≥ pT2, as previously reported^7^. In addition, normal bladder tissue (T2234040, BCH, Newark, CA) was recruited for the control.

The study was conducted in accordance with the principles of the Declaration of Helsinki. The human ethics board of each institution approved this study, and written informed consent was obtained from all patients prior to enrollment (Iwate Medical University; protocol No. 2019-083. The Cancer Institute Hospital of Japanese Foundation for Cancer Research; protocol No. 2021-GA-1096).

### Multiplex fluorescence immunohistochemistry

As previously described^7^, four-micrometer-thick tissue sections obtained from formalin-fixed paraffin-embedded (FFPE) blocks were stained via mFIHC with an Opal IHC kit (AKOYA Biosciences, CA, USA). One representative FFPE block was selected from the pre-NAC and post-NAC specimens per case by the pathologist. The antibodies, dilutions, and activation conditions used are listed in Table S1. Next, a whole slide was scanned using an automated imaging system (Vectra ver. 3.0, AKOYA Biosciences). The whole specimens were captured, with an average of 20 areas at × 200 magnification. We segmented tumor tissues into cancer cell nests and stromal resions, and identified each stained cell with specific phenotypes using image-analyzing software (InForm, AKOYA Biosciences). Before the final evaluation, manual training sessions for phenotype recognition were conducted, followed by automatic machine learning for the algorithm. Two researchers (Ikarashi D, and S.T.) independently evaluated the stained slides, found no significant difference in result. An analytic program (Spotfire, TIBCO software, CA, USA) counted the infiltrating immune cells with specific phenotypes per mm^2^ in cancer cell nests (intratumor) plus stromal regions (stroma) (Supplemental Fig. 2).

### Statistical analysis

The densities of immune cells between pre- and post-NAC tissue were compared with Wilcoxon signed rank test. Progression-free survival (PFS) and cancer-specific survival (CSS) rates were calculated using the Kaplan–Meier method. PFS was defined as the time from the radical cystectomy to radiographic or clinical progression. CSS was defined as the time from the radical cystectomy to cancer-specific death or loss-to-follow-up censoring. All statistical analyses were performed using JMP 14.0 software (SAS Institute Inc., Cary, NC, USA). Differences with *p* < 0.05 were considered statistically significant for all statistical comparisons.

### Ethics approval and consent to participate

The study was conducted in accordance with the principles of the Declaration of Helsinki. The human ethics board of each institution approved this study, and written informed consent was obtained from all patients prior to enrollment (Iwate Medical University; Protocol No. 2019–083, The Cancer Institute Hospital of Japanese Foundation for Cancer Research; protocol No. 2021-GA-1096).

## Results

### Patient characteristics

**Figure 1:**
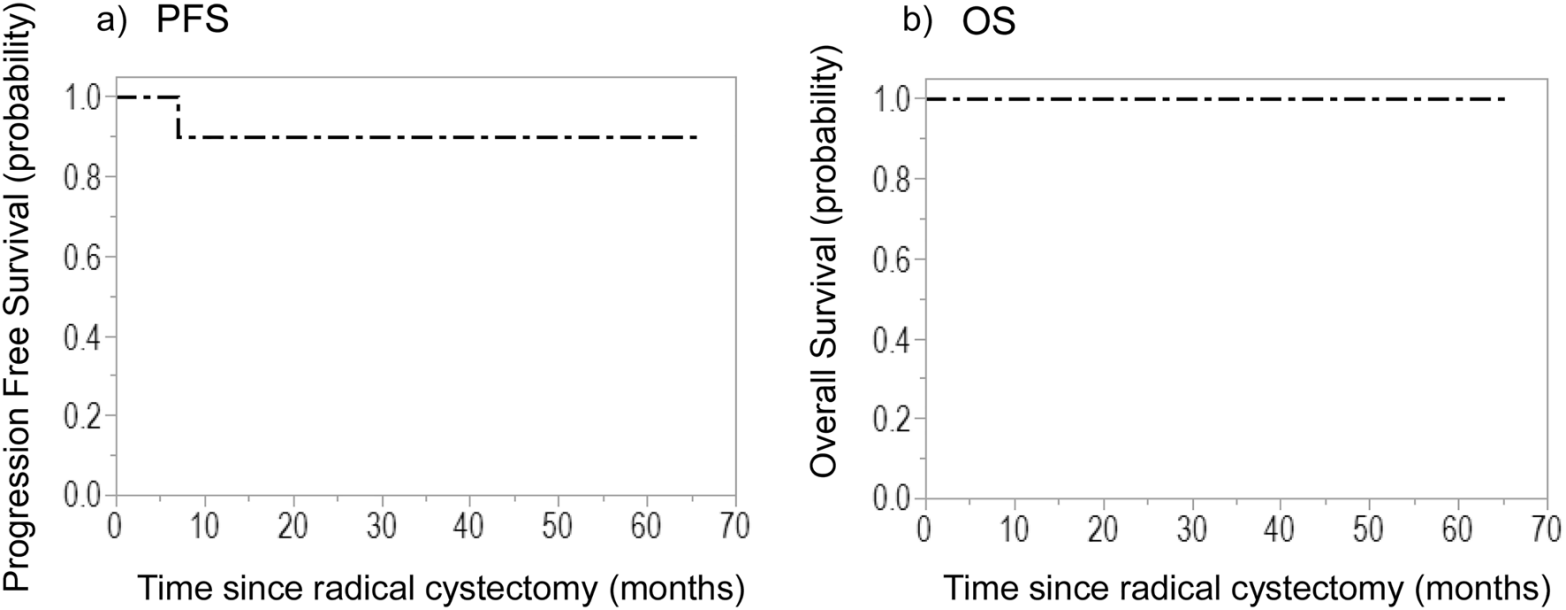
Kaplan–Meier curves of (**a**) PFS and (**b**) OS of patients with pathological complete response treated with neoadjuvant chemotherapy for muscle-invasive bladder cancer.

The clinical and pathological features of the patients with pCR are described in Table 1. The median follow-up duration after radical cystectomy was 27.6 (range, 3.1–65.6) months. The 5-year PFS and CSS were 90% and 100%, respectively (Fig. 1), indicating a strong association between pCR and patient prognosis. Only one patient had postoperative recurrence. However, in this case, the recurrence site was in the urethra.

**Table 1:** Patients characteristics.

Variable	Level	All patients (n = 51)	pCR patients (n = 12)
Age	median	68 (43–78)	67 (43–74)
Sex	Male	38 (74.5%)	10 (83.8%)
	Female	13 (25.5%)	2 (16.7%)
Smoking	Yes	30 (58.8%)	9 (75%)
	No	13 (25.5%)	0
	Unknown	8 (15.7%)	3 (25%)
Clinical stage	cT2	30 (58.8%)	9 (75%)
	cT3	20 (39.2%)	3 (25%)
	cT4	1 (2%)	0
Histology	Pure UC	49 (96.1%)	11 (91.6%)
	UC + variant histology	2 (3.9%)	1 (8.4%)
TURBT	Yes	17 (33.3%)	10 (83.3%)
	No	34 (66.7%)	2 (16.7%)
NAC regimen	GC/CaG	40 (78.4%)	10 (83.3%)
	MVAC	11 (21.6%)	2 (16.7%)
Pathological stage	pT0	12 (23.5%)	12 (100%)
	pTis/1	17 (33.4%)	0
	pT2	10 (19.6%)	0
	≥ pT3	12 (23.5%)	0

UC, urotherial carcinoma; TURBT, trans urethral resection of bladder cancer; NAC, neoadjuvant chemotherapy; GC, gemcitabine + cisplatin; CaG, carboplatin + gemcitabine; MVAC, methotrexate + vinblastine + doxorubicin (adriamycin) + cisplatin.

### Changes in immune cells before and after NAC in pCR patients

**Figure 2:**
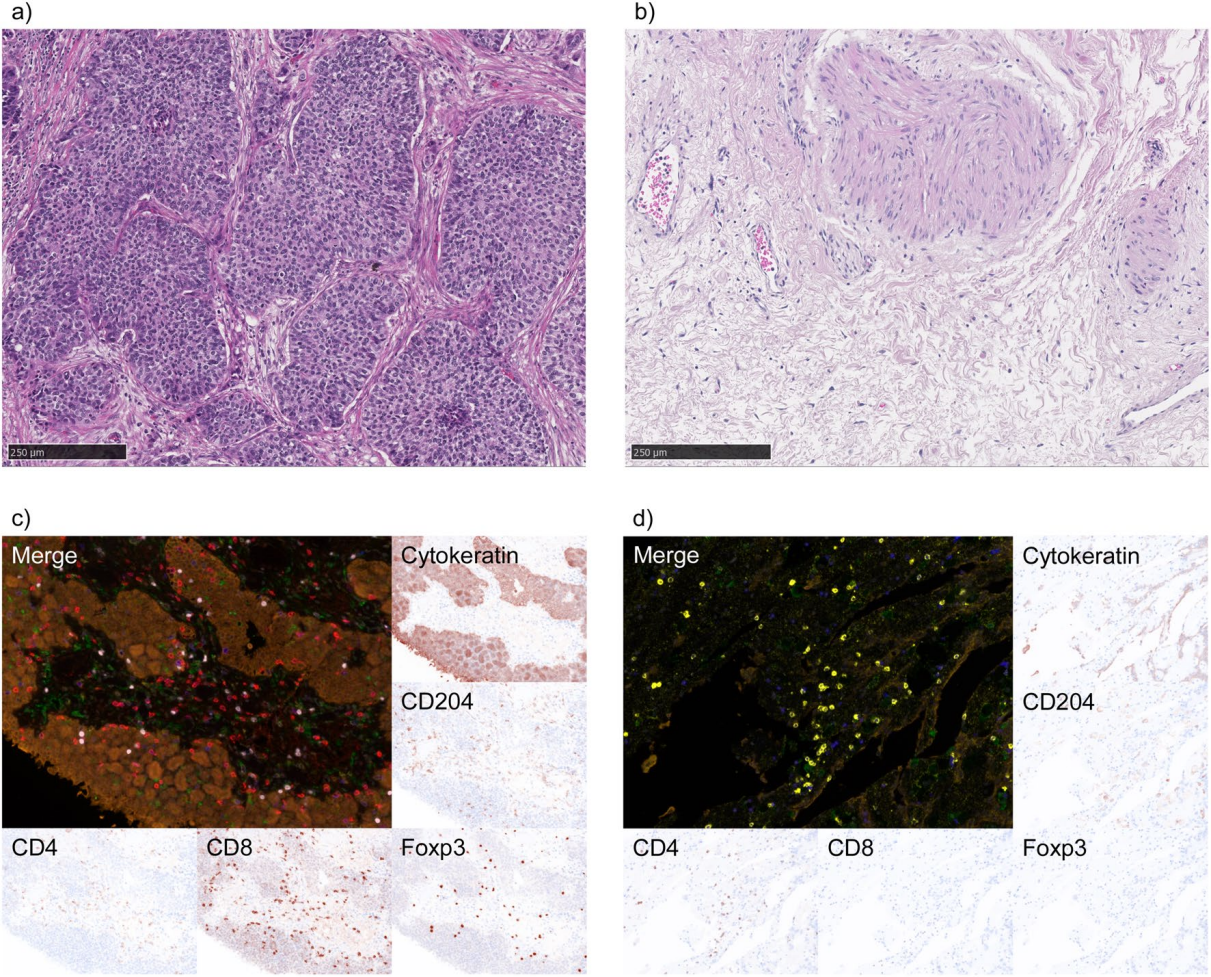
Representative image of hematoxylin & eosin staining of (**a**) pre-NAC tissue and (**b**) post-NAC tissue in a patient who achieved pathological complete response. Representative image of multiplex fluorescent immunohistochemistry of (**c**) pre-NAC and (**d**) post-NAC tissue. Multiplex fluorescent immunohistochemistry was performed using the following antibodies against CD3 (blue), CD4 (yellow), CD8 (red), Foxp3 (pink), CD204 (green), and pan-cytokeratin (brown). NAC; neoadjuvant chemotherapy.

**Figure 3:**
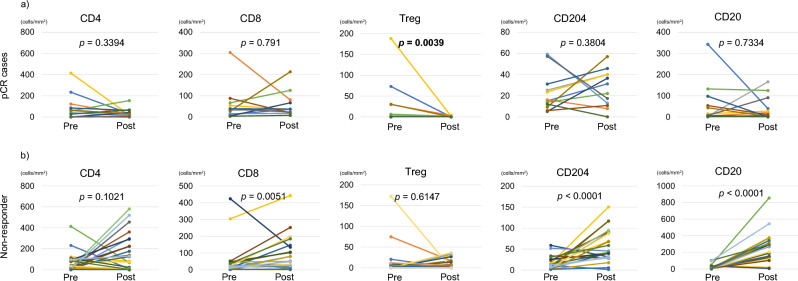
Relationship between the density of each immune cell type in pre-NAC and post-NAC tissue in (**a**) pathological complete response (pCR) and (**b**) non-responder groups, as follows: CD4, CD8, Treg, CD204, and CD20.

We assessed the infiltration of various immune cells on pre- and post-NAC specimens via hematoxylin/eosin staining and mFIHC (Fig. 2). We observed significantly decreased levels of CD3^+^CD4^+^Foxp3^+^ T cells in the post-NAC tissue compared with the pre-NAC tissue (Fig. 3a; *p* = 0.0039). However, we observed no significant difference in CD4^+^T cells, CD8^+^ T cells, CD204^+^ cells, and CD20^+^ cells between the pre- and post-NAC tissue (Fig. 3a).

### Comparison of change in regulatory T cells (Tregs) between pCR and NAC non-responders

**Figure 4:**
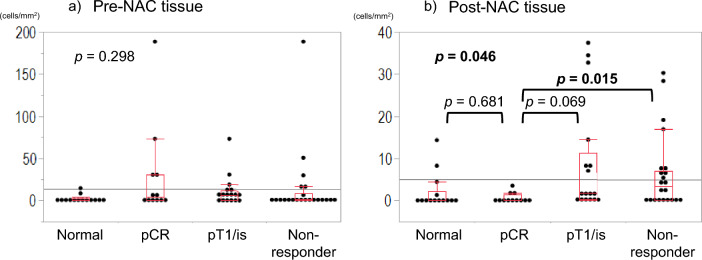
Comparison of the densities of Tregs between normal bladder tissue, pCR cases, pT1/is cases and non-responder groups in pre- and post-NAC tissue.

We analyzed the changes in CD3^+^CD4^+^Foxp3^+^ T cells before and after treatment in the NAC non-responder group (≥ pT2) using the same analytical conditions without tissue segmentation as previously reported^7^. In the non-responder group, we observed no significantly decreased levels of CD4^+^Foxp3^+^ T cells in the post-NAC tissue compared with the pre-NAC tissue (Fig. 3b; *p* = 0.6147, Supplemental table 2). Furthermore, we compared the densities of Treg between normal bladder tissue, pre- and post-treatment tissue in pCR cases, pre- and post-treatment tissue in pT1/is as partial response cases and pre- and post-treatment tissue in non-responder groups. In pre-NAC tissue, there was no significant difference of the Tred densities between each group (Fig. 4a; *p* = 0.298). In post-NAC tissue, the Treg densities were significantly higher in the non-responder group than in the pCR group (Fig. 4b; *p* = 0.015), and the Treg densities were tend to higher in pT1/is group than in the pCR group (Fig. 4b; *p* = 0.069). While there was no significant difference between pCR cases and normal bladder (Fig. 4b; *p* = 0.618).

## Discussion

In this study, we demonstrated that only the densities of CD3^+^CD4^+^Foxp3^+^ T cells (Tregs) on the tumor bed were significantly decreased and almost disappeared in post-NAC tissue compared with pre-NAC tissue in those with pCR. However, almost all immune cells, including Tregs, were not significantly decreased in post-NAC tissue compared with pre-NAC tissue in non-responders. This result might indicate that the disappearance of the tumor terminated the immune response, and that the Tregs which appeared after starting immune response and exerted a critical brake on immune response of T and B cells in the TiME were no longer needed. Therefore, the densities of Tregs in pCR cases were similar to those of normal bladder tissue. Furthermore, the immune response was continued in the microenvironment, including suppressive immune cells in non-responder groups with residual tumor. Tregs in the tumor may reflect tumor volume in the TiME. In previous reports, surgical resection of the primary tumor may restore tumor immune response via reversal of immune suppression^13,15^. Our results also suggest that chemotherapy may have restored the tumor immune response as well as surgical resection by achieving a pCR.

Tregs are an immunosuppressive subset of CD4^+^ T cells first identified by Sakaguchi et al. in 1995 as CD25^+^CD4^+^ T cells^16^. Tregs also maintain immune homeostasis, support self-tolerance, and suppress excessive immune responses. Moreover, infiltrating Tregs in the TiME suppress the antitumor immune response driven by CD8^+^ T cells, promoting tumor development and growth^17,18^. Tumor-infiltrating Tregs have also been reported as prognostic markers in various native cancers^18^. Our results suggest that the marked reduction of Treg in TiME led to a favorable prognosis. Sun et al.^19^ reported that CD3^high^FoxP3^−^ TILs had a better prognosis compared to CD3^high^FoxP3^+^ TILs or CD3^low^FoxP3^−^ TILs (*p* = 0.0035) in patients with urothelial carcinoma in bladder. Furthermore, we previously reported that the intratumoral CD204^+^ cells as M2 macrophages were significantly increased in post-NAC tissue compared with pre-NAC tissue in the non-responder group^7^. In the present study, we observed that CD204^+^ cells were not significantly increased in post-NAC tissue compared with pre-NAC tissue in pCR patients. Moreover, we observed that Tregs were not significantly decreased in post-NAC tissue compared with pre-NAC tissue in non-responder groups. These results indicate that a decrease in Tregs and CD204^+^ cells with tumor volume reduction, which suppress antitumor immune response within the tumor microenvironment, is related to the prognosis of pCR cases. In addition, we have experience with pCR cases after pembrolizumab treatment in chemo-resistant UTUC patient, and analysis of the tumor microenvironment in these pCR specimens showed increasing CD8^+^ T cell and CD20^+^ cell^20^, suggesting that immune checkpoint inhibitors and platinum drugs may alter the tumor microenvironment differently.

This study has some limitations. First, the associations with the status of Tregs in the TiME and in peripheral blood were unknown because of the study was retrospective. Second, a conclusion could not be drawn in this case series because of the relatively small number of patients. A recent review indicated that the association between the TiME and the systemic tumor immune environment is very complex, involves various pathways and mechanisms, and varies with the primary tumor types^21^. In addition, Tregs are also present in the systemic tumor immune environment at a level similar, higher, or lower relative to the TiME in NSCLC, HCC, and NPC^21^. A future prospective study with a large cohort through multi-center study and evaluation of peripheral blood would validate the results of this exploratory study. Even with these considerations, our findings showed the possibility of reduced Tregs upregulating the immune response and therefore being associated with complete response to cisplatin in a small number of cases. Thus, we suggest that our findings have the potential to lead the clinical strategies to deplete Treg cells and control the Treg cell function to enhance anti-tumor immune responses. Third, it is difficult to distinguish the suppressive function of tumor-infiltrating Tregs based on Foxp3 expression alone^22^. Furthermore, Foxp3 is not only expressed in Tregs but also weakly in activated T cells, and it would be difficult to distinguish these subpopulations by immunohistochemistry, in which quantitative evaluation of antigen expression levels is difficult^23^. Therefore, we only evaluated Foxp3 expression in CD4^+^ T cells without a functional marker of Treg. Tregs are composed of functionally distinct subpopulations, including naive Tregs, effector Tregs, and non-suppressive Tregs. We believe this study is valuable because we could make immunological evaluations in TiME before and after preoperative chemotherapy in detail by mFIHC using actual clinical tumor specimens. Our results may support combination therapeutic strategies aimed at Treg removal in neoadjuvant and adjuvant settings near future.

## Conclusion

This is the first report to demonstrate that change in immune cells in the TiME was related to pCR in those with MIBC in the platinum-based NAC setting. In patients with pCR, the immune response in the tumor microenvironment were observed to be temporarily arrested with tumor shrinkage after NAC, and Tregs were the main component of these immune responses. As a result, Tregs are significantly decreased from tumor microenvironment in pCR patients, which might improve the tumor immune response and result in a favorable prognosis.

### Supplementary Information

Supplementary Legends.

Supplementary Figures.

Supplementary Table S1.

Supplementary Table S2.

Supplementary Table S3.

## Data Availability

All data supporting the results are presented with results, in the figures, and supplemental materials.

## References

[1] Rosenblatt R, Sherif A, Rintala E, Wahlqvist R, Ullén A, Nilsson S (2012). Pathologic downstaging is a surrogate marker for efficacy and increased survival following neoadjuvant chemotherapy and radical cystectomy for muscle-invasive urothelial bladder cancer. Eur. Urol..

[2] Winoker JS, Liaw CW, Galsky MD, Wiklund P, Mehrazin R (2020). Clinical complete response after neoadjuvant chemotherapy for muscle-invasive bladder cancer: A call for standardized assessments and definitions. Eur. Urol. Focus.

[3] Grossman HB, Natale RB, Tangen CM, Speights VO, Vogelzang NJ, Trump DL (2003). Neoadjuvant chemotherapy plus cystectomy compared with cystectomy alone for locally advanced bladder cancer. N. Engl. J. Med..

[4] Winquist E, Kirchner TS, Segal R, Chin J, Lukka H, Genitourinary Cancer Disease Site Group (2004). Neoadjuvant chemotherapy for transitional cell carcinoma of the bladder: A systematic review and meta-analysis. J. Urol..

[5] Pfister C, Gravis G, Fléchon A, Soulié M, Guy L, Laguerre B (2021). Randomized Phase III trial of dose-dense methotrexate, vinblastine, doxorubicin, and cisplatin, or gemcitabine and cisplatin as perioperative chemotherapy for patients with muscle-invasive bladder cancer. Analysis of the GETUG/AFU V05 VESPER trial secondary endpoints: Chemotherapy toxicity and pathological responses. Eur. Urol..

[6] Lv B, Wang Y, Ma D, Cheng W, Liu J, Yong T (2022). Immunotherapy: Reshape the tumor immune microenvironment. Front. Immunol..

[7] Ikarashi D, Kitano S, Tsuyukubo T, Takenouchi K, Nakayama T, Onagi H (2022). Pretreatment tumour immune microenvironment predicts clinical response and prognosis of muscle-invasive bladder cancer in the neoadjuvant chemotherapy setting. Br. J. Cancer.

[8] Jiang Y, Zhang Q, Hu Y, Li T, Yu J, Zhao L (2018). ImmunoScore signature: A prognostic and predictive tool in gastric cancer. Ann. Surg..

[9] Nishino M, Ramaiya NH, Hatabu H, Hodi FS (2017). Monitoring immunecheckpoint blockade: Response evaluation and biomarker development. Nat. Rev. Clin. Oncol..

[10] Seiler R, Gibb EA, Wang NQ, Oo HZ, Lam HM, van Kessel KE (2019). Divergent biological response to neoadjuvant chemotherapy in muscle-invasive bladder cancer. Clin. Cancer Res..

[11] Qian LW, Fourcaudot AB, Yamane K, You T, Chan RK, Leung KP (2016). Exacerbated and prolonged inflammation impairs wound healing and increases scarring. Wound Repair. Regen..

[12] Schreiber, H. Tumor immunology. In *Fundamental Immunology* (ed. Paul, W. E.) 1557–1591 (Lippincott Williams & Wilkins Baltimore, 2003).

[13] Salvadori S, Martinelli G, Zier K (2000). Resection of solid tumors reverses T cell defects and restores protective immunity. J. Immunol. Baltim. Md. 1950.

[14] Stack EC, Wang C, Roman KA, Hoyt CC (2014). Multiplexed immunohistochemistry, imaging, and quantitation: A review, with an assessment of Tyramide signal amplification, multispectral imaging and multiplex analysis. Methods.

[15] Danna EA, Sinha P, Gilbert M, Clements VK, Pulaski BA, Ostrand-Rosenberg S (2004). Surgical removal of primary tumor reverses tumor-induced immunosuppression despite the presence of metastatic disease. Cancer Res..

[16] Sakaguchi S, Sakaguchi N, Asano M, Itoh M, Toda M (1995). Immunologic self-tolerance maintained by activated T cells expressing IL-2 receptor alpha-chains (CD25). Breakdown of a single mechanism of self-tolerance causes various autoimmune diseases. J. Immunol..

[17] Rabinovich GA, Gabrilovich D, Sotomayor EM (2007). Immunosuppressive strategies that are mediated by tumor cells. Annu. Rev. Immunol..

[18] Itahashi K, Irie T, Nishikawa H (2022). Regulatory T-cell development in the tumor microenvironment. Eur. J. Immunol..

[19] Sun ZJ, Zhao JW, Zhao M, Chen Y, Zhang X, Li HC (2022). CD3high and FoxP3- tumor-infiltrating lymphocytes in the invasive margin as a favorable prognostic marker in patients with invasive urothelial carcinoma of the bladder. Anti Cancer Drugs.

[20] Ikarashi D, Kitano S, Ishida K, Nakatsura T, Shimodate H, Tsuyukubo T (2020). Complete pathological response to neoadjuvant pembrolizumab in a patient with chemoresistant upper urinary tract urothelial carcinoma: A case report. Front. Oncol..

[21] Xu L, Zou C, Zhang S, Chu TSM, Zhang Y, Chen W (2022). Reshaping the systemic tumor immune environment (STIE) and tumor immune microenvironment (TIME) to enhance immunotherapy efficacy in solid tumors. J. Hematol. Oncol..

[22] Saito T, Nishikawa H, Wada H, Nagano Y, Sugiyama D, Atarashi K (2016). Two FOXP3(+)CD4(+) T cell subpopulations distinctly control the prognosis of colorectal cancers. Nat. Med..

[23] Takeuchi Y, Nishikawa H (2016). Roles of regulatory T cells in cancer immunity. Int. Immunol..

