# Supplementary material for: Pathological complete response to neoadjuvant chemotherapy may improve antitumor immune response via reduction of regulatory T cells in muscle-invasive bladder cancer
Source: Sci Rep. 2024 Jan 16;14:1442. doi: 10.1038/s41598-024-51273-7 (PMC10792090; doi:10.1038/s41598-024-51273-7)
Supplement: Supplementary file 1 — Supplementary Legends. [file 41598_2024_51273_MOESM1_ESM.docx]

**Additional File**

Pathological complete response to neoadjuvant chemotherapy may improve antitumor immune response via reduction of regulatoty T cells in muscle-invasive bladder cancer.

Daiki Ikarashi^a,b^, Shigehisa Kitano^b^, Takashi Tsuyukubo^a^, Makiko Yamashita^b^, Tomohiko Matsuura^a^ Shigekatsu Maekawa^a^, Renpei Kato^a^, Yoichiro Kato^a^, Mitsugu Kanehira^a^, Ryo Takata^a^, Tamotsu Sugai^c^, Wataru Obara^a^

^a^ Department of Urology, Iwate Medical University School of Medicine, Iwate 028-3695, Japan

^b^ Division of Cancer Immunotherapy Development, Department of Advanced Medical Development, The Cancer Institute Hospital of Japanese Foundation for Cancer Research, Tokyo 135-8550, Japan

^c^ Department of Pathology, Iwate Medical University School of Medicine, Iwate 028-3695, Japan

**List of Supplemental Materials**

**Supplemental table 1.** List of antibodies

**Supplemental table 2.** The change in densities of each immune cell between pre-NAC and post-NAC tissue in pCR and non-responder groups. In pCR cases, only regulatory T cells were significantly decreased in post-NAC tissue compared with pre-NAC tissue.

**Supplemental table 3.** The densities of each immune cell of all cases used for statistical analysis.

**Supplemental Figure 1.**

The flow diagram of patients included in the study.

**Supplemental Figure 2.**

Representative image of immunofluorescence in bladder cancer with the following markers: CD3 (blue), CD4 (yellow), CD8 (red), FoxP3 (pink), CD204 (green), and cytokeratin (brown) (a). Tissue segmentation of the intratumoral (red), stromal (green) areas, and other areas (blue) (b). Cell segmentation (c) and cell phenotyping (d) demonstrated infiltrating immune cells in the intratumoral and peritumoral areas (cancer cells, orange; CD4+ T cells, yellow; CD8+ T cells, red; CD204+ cells, green; stromal cells, gray).

**Supplemental Figure 3.**

The Changes in immune cells before and after NAC specimen in pT1/is patient group.
