# Supplementary material for: Pathological complete response to neoadjuvant chemotherapy may improve antitumor immune response via reduction of regulatory T cells in muscle-invasive bladder cancer
Source: Sci Rep. 2024 Jan 16;14:1442. doi: 10.1038/s41598-024-51273-7 (PMC10792090; doi:10.1038/s41598-024-51273-7)
Supplement: Supplementary file 2 — Supplementary Figures. [file 41598_2024_51273_MOESM2_ESM.pptx]

## Slide 1
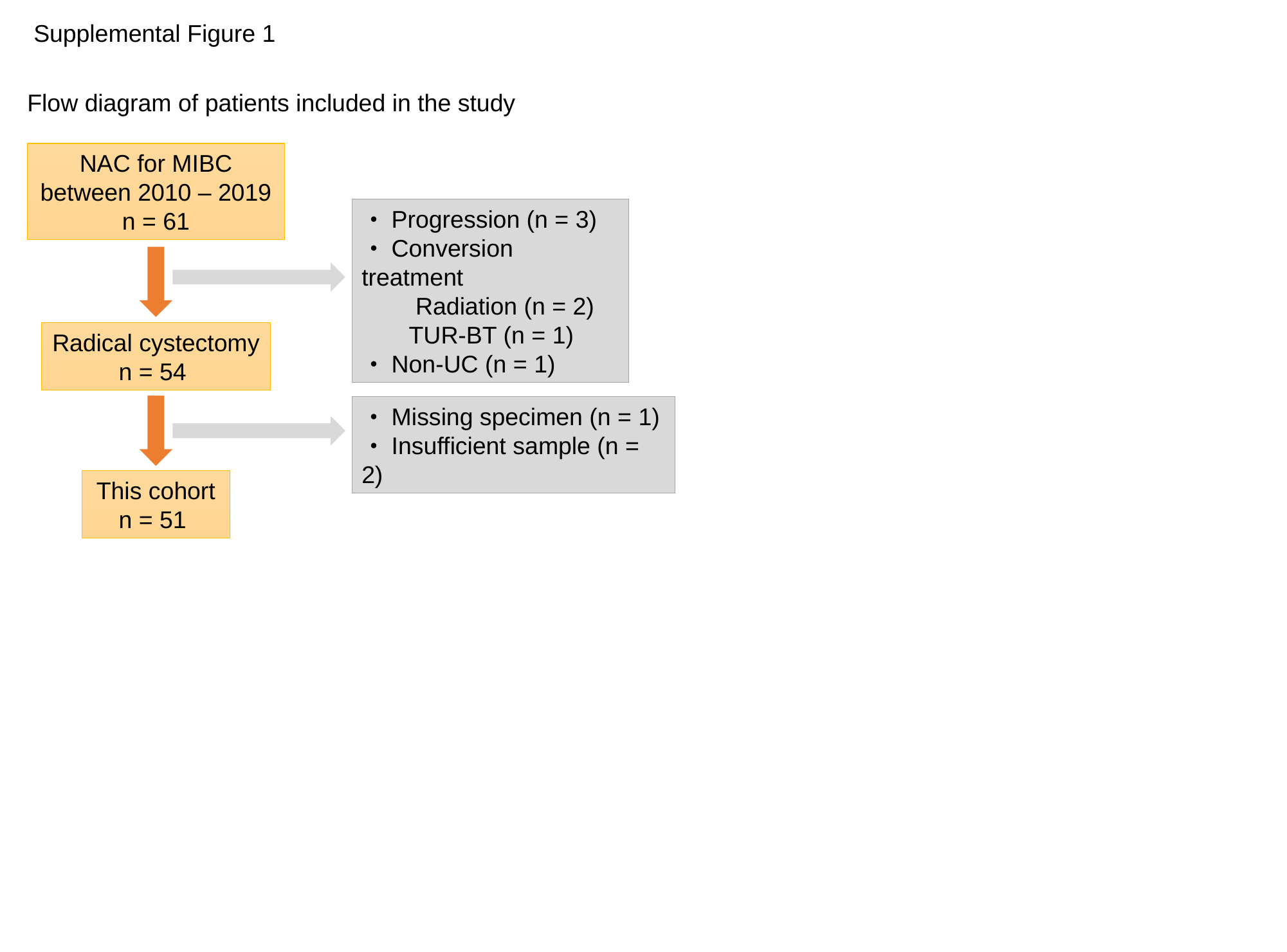

Supplemental Figure 1
Flow diagram of patients included in the study
NAC for MIBC
between 2010 – 2019
 n = 61
・Progression (n = 3)
・Conversion treatment
　　Radiation (n = 2)
 TUR-BT (n = 1)
・Non-UC (n = 1)
Radical cystectomy
n = 54
・Missing specimen (n = 1)
・Insufficient sample (n = 2)
This cohort
n = 51

## Slide 2
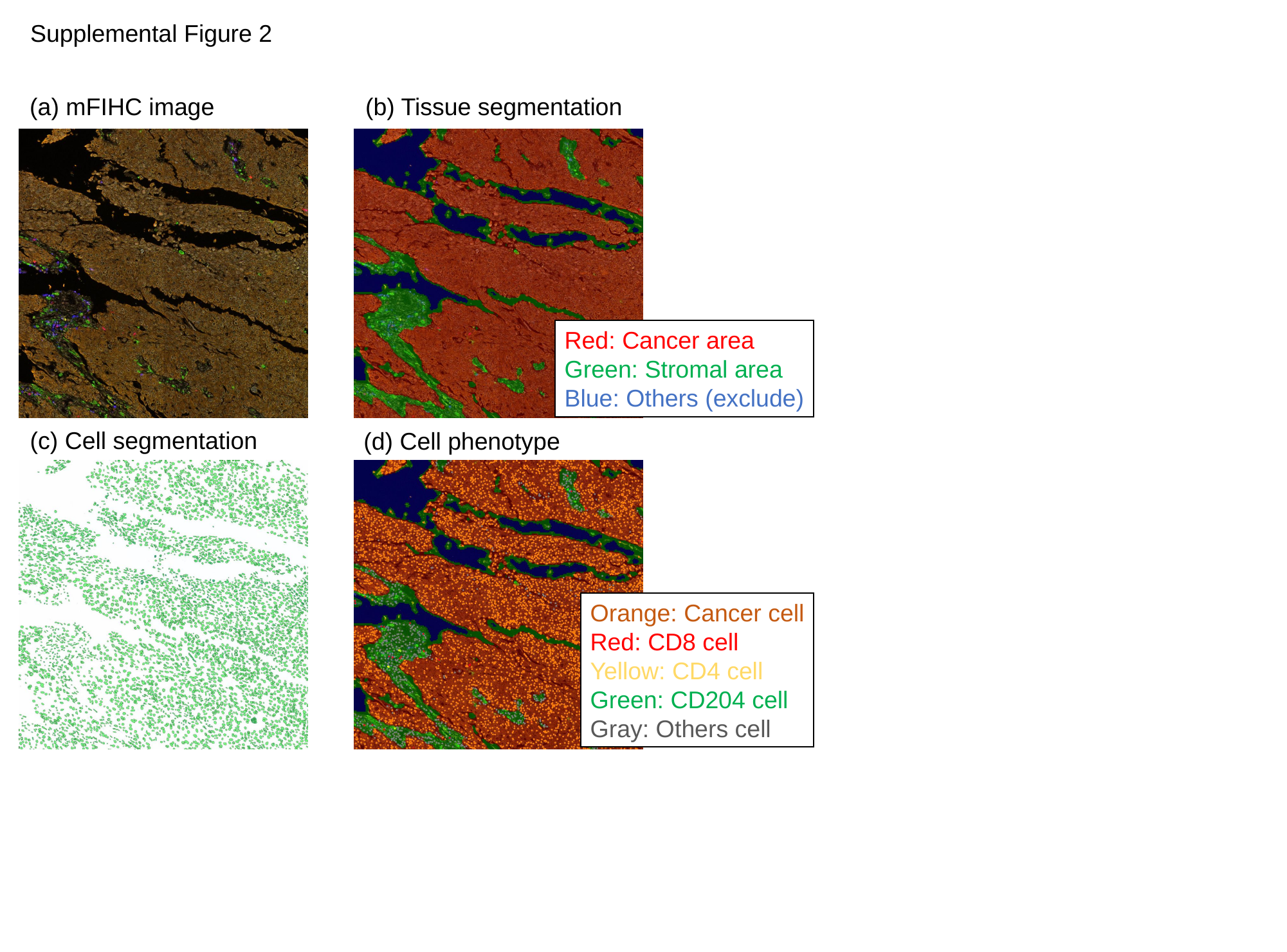

Supplemental Figure 2
(a) mFIHC image
(b) Tissue segmentation
Red: Cancer area
Green: Stromal area
Blue: Others (exclude)
(c) Cell segmentation
(d) Cell phenotype
Orange: Cancer cell
Red: CD8 cell
Yellow: CD4 cell
Green: CD204 cell
Gray: Others cell

## Slide 3
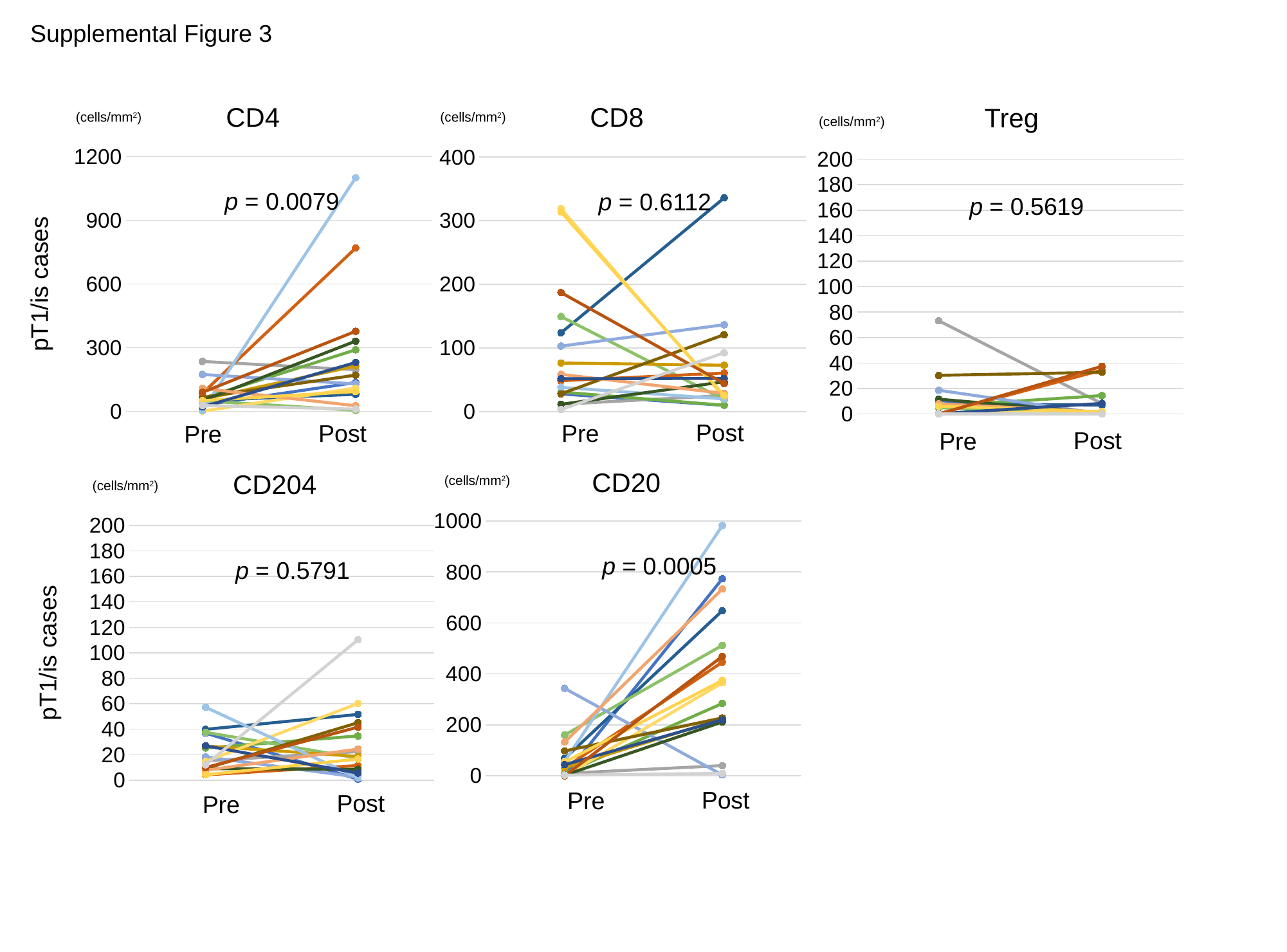

Supplemental Figure 3
CD4
CD8
Treg
(cells/mm2)
(cells/mm2)
(cells/mm2)
### Chart
| Category | | | | | | | | | | | | | | | | | | | | | | | | | | | | | | | | | | | | | | | | | | | | | | | | | | | |
|---|---|---|---|---|---|---|---|---|---|---|---|---|---|---|---|---|---|---|---|---|---|---|---|---|---|---|---|---|---|---|---|---|---|---|---|---|---|---|---|---|---|---|---|---|---|---|---|---|---|---|---|
### Chart
| Category | | | | | | | | | | | | | | | | | | | | | | | | | | | | | | | | | | | | | | | | | | | | | | | | | | | |
|---|---|---|---|---|---|---|---|---|---|---|---|---|---|---|---|---|---|---|---|---|---|---|---|---|---|---|---|---|---|---|---|---|---|---|---|---|---|---|---|---|---|---|---|---|---|---|---|---|---|---|---|
### Chart
| Category | | | | | | | | | | | | | | | | | | | | | | | | | | | | | | | | | | | | | | | | | | | | | | | | | | | |
|---|---|---|---|---|---|---|---|---|---|---|---|---|---|---|---|---|---|---|---|---|---|---|---|---|---|---|---|---|---|---|---|---|---|---|---|---|---|---|---|---|---|---|---|---|---|---|---|---|---|---|---|p = 0.0079
p = 0.6112
p = 0.5619
pT1/is cases
Post
Post
Pre
Pre
Post
Pre
CD20
CD204
(cells/mm2)
(cells/mm2)
### Chart
| Category | | | | | | | | | | | | | | | | | | | | | | | | | | | | | | | | | | | | | | | | | | | | | | | | | | | |
|---|---|---|---|---|---|---|---|---|---|---|---|---|---|---|---|---|---|---|---|---|---|---|---|---|---|---|---|---|---|---|---|---|---|---|---|---|---|---|---|---|---|---|---|---|---|---|---|---|---|---|---|
### Chart
| Category | | | | | | | | | | | | | | | | | | | | | | | | | | | | | | | | | | | | | | | | | | | | | | | | | | | |
|---|---|---|---|---|---|---|---|---|---|---|---|---|---|---|---|---|---|---|---|---|---|---|---|---|---|---|---|---|---|---|---|---|---|---|---|---|---|---|---|---|---|---|---|---|---|---|---|---|---|---|---|p = 0.0005
p = 0.5791
pT1/is cases
Post
Pre
Post
Pre
